# Pulsatile varicose veins simulating femoral artery aneurysm: case report

**DOI:** 10.1590/1677-5449.200070

**Published:** 2021-06-11

**Authors:** Cleilson Almeida Marchesi, Márcia Porto Assis

**Affiliations:** 1 Universidade Federal do Espírito Santo – UFES, Hospital Universitário Cassiano Antonio Moraes – HUCAM, Vitória, ES, Brasil.

**Keywords:** varicose veins, pulsatile, tricuspid regurgitation, vascular ultrasound

## Abstract

Severe tricuspid regurgitation is mentioned as a factor associated with development or recurrence of varicose veins in the lower limbs and may present with retrograde pulsatile flow. Differential etiological diagnosis of this ultrasound finding must include investigation of arteriovenous fistulas, since the treatment methods are different. Given the complexity of the general condition of patients with tricuspid regurgitation, treatment for pulsatile varices should be chosen on a case-by-case basis after multidisciplinary evaluation. All of the techniques commonly used to treat varicose veins are part of the therapeutic arsenal, as well as combinations of them, taking into account the severity of clinical manifestations and the cardiovascular risk involved. We report a case of pulsatile varices secondary to tricuspid regurgitation diagnosed when investigating a primary suspicion of femoral artery aneurysm in a 73-year-old patient, CEAP 4a, oligosymptomatic, who was treated with postural measures and elastic compression.

## INTRODUCTION

The prevalence of peripheral vascular diseases tends to increase as people age and it is relatively frequent for peripheral vascular diseases and cardiovascular diseases to coexist.^1^ Severe tricuspid regurgitation (TR) is cited as a factor that is associated with development or relapse of lower limb varicose veins, which can exhibit pulsating, retrograde flow.^2^

Although recognition of pulsatility in peripheral veins caused by TR and valve incompetence has been described since 1827,^3^ even with current widespread use of Doppler ultrasound, ignorance of the pattern of these changes is reflected in the poor or absent descriptions in examination reports.^4^ We report a case of pulsatile varicose veins (PVV) in a patient with TR. The study was submitted to the Ethics Committee (CAAE 36635420.1.0000.50.71) and approved in ruling number 4.426.72.

## CASE REPORT

The patient was a 73-year-old female, with hypertension, grade C heart failure (HF), and congestive liver disease, who presented at the emergency room of a University Hospital complaining of nocturnal paroxysmal dyspnea and hemoptysis. Transthoracic echocardiogram showed a thickened mitral valve, with significant insufficiency, and reversed systolic flow in the left superior pulmonary vein; tricuspid valve closure was dysfunctional and there was important dilation of the right atrium (56 mm) and inferior vena cava (> 21 mm in diameter, with < 50% variability during respiration).

Physical examination at first presentation detected symmetrical edema of the lower limbs, large caliber varicose veins, discrete ochrodermatitis involving the distal third of the legs, although with no eczemas or ulcerations (CEAP clinical venous disease classification was 4a). There was a notable pulsating swelling in the left inguinal region.

**Figure 1:**
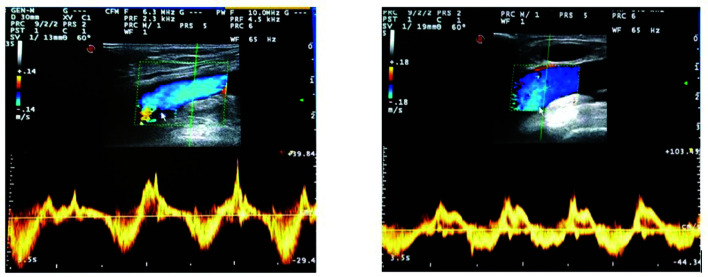
Bidirectional pulsating flow in left and right common femoral veins.

**Figure 2:**
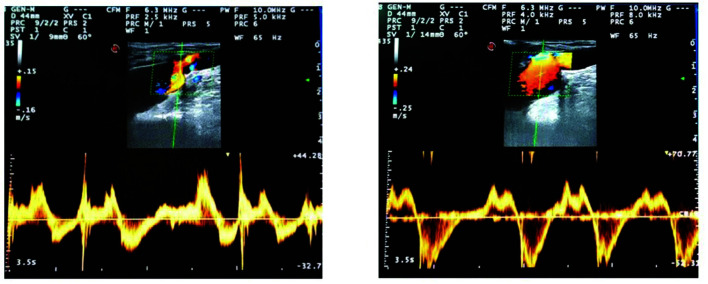
Bidirectional flow at left and right saphenofemoral junctions.

**Figure 3:**
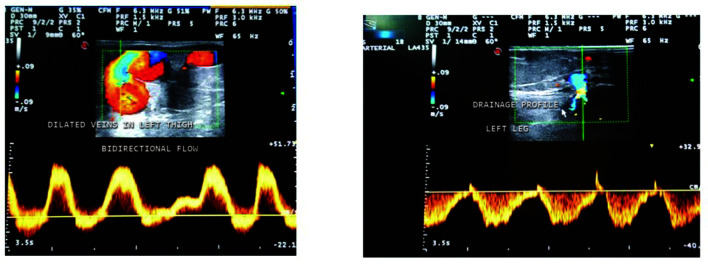
Bidirectional flow in varicose veins of the left thigh and perforator vein in the leg.

Pursuing a hypothesis of femoral artery aneurysm concomitant with the varicosities, arterial Doppler ultrasonography of the left lower limb was requested. This showed triphasic flow in all segments investigated, with no significant parietal changes. Direct and indirect echographic signs of arteriovenous communication (fistula) were absent. During the venous study, alternating (bidirectional) flow was observed in the left common femoral vein and saphenofemoral junction. Supplementary venous studies of both limbs found no signs of deep venous thrombosis, but revealed an alternating spectral curve in the right deep venous axis, extending to both saphenofemoral junctions and great saphenous veins and also present in varicose dilatations in the thighs. On the left, in the territory of the inguinal swelling, large caliber varicose veins crossed anterior to the femoral artery, while the varicose tributaries that extended along the thigh and knee communicated with a perforating vein on the medial surface of the proximal third of the leg, and the perforator exhibited an alternating spectral curve with predominant blood flow in the direction of the deep vein system (Figures 1, 2, and 3).

After the ultrasonographic diagnosis, an assessment in conjunction with the vascular surgery team was requested, and this physical examination revealed pulsatility along the entire superficial venous axis during, in addition to absence of thrill on palpation and slow filling of varicosities during the compression/decompression maneuver. Clinical treatment was chosen, with postural measures and elastic compression. Over the course of her stay, the patient exhibited significant clinical improvement of her heart disease and limb edema. Since this was oligosymptomatic varicose disease, with severe, recently compensated cardiac comorbidity in a patient of advanced age, the decision was taken to maintain ambulatory clinical treatment, with no invasive interventions. During the subsequent months, she suffered several additional episodes of decompensated HF and recurrent hospital admissions and died after 1 year of follow-up.

## DISCUSSION

In physiological conditions, venous flow observed with Doppler ultrasonography close to the heart exhibits a multiphase wave with two anterograde and two retrograde components. The first and widest anterograde component corresponds to negative atrioventricular septum pressure during systole and is followed by a retrograde component, which represents the positive atrial pressure due to atrial flow. A further positive deflection occurs when the tricuspid valve opens, which is then followed by the last negative deflection, produced by atrial contraction.^5^ As the examination proceeds away from the heart in the direction of the lower limbs, the high complacency and capacitance of these territories attenuate the pulsatility and flow comes into phase with respiration.^5^

In the presence of severe TR, pulsating flow with a retrograde component can be found in the middle suprahepatic vein and, more rarely, in the lower limbs.^5^ According to a study by Ribeiro et al.,^5^ presence of pulsatility in the femoral veins can be observed in around 15.2% of the population of people over the age of 60 years, and has an important correlation with tricuspid insufficiency. In the presence of an incompetent saphenofemoral junction, “ventricularization” of flow in the deep vein system caused by significant TR can be transferred to the saphenous axis and to lower limb varicosities.^1^^,^ 6 ^,^ 7 In the great majority of cases, the changes caused by cardiac effects on the flow waveforms in peripheral vessels go unrecognized, and even when they are observed, they are rarely noted in the report.^4^

Arteriovenous fistula should be investigated during differential diagnosis of findings of venous system pulsatility, although it is unlikely if the findings are bilateral.^6^^,^ 8 Klein et al.^9^ reported a case of unilateral PVV that developed 15 years after mitral valvuloplasty with intraoperative cardiopulmonary bypass. In this case, development of the varicose condition ipsilateral to manipulation of the femoral vessels added weight to the hypothesis of iatrogenic arteriovenous fistula, prompting surgical exploration of the femoral artery up to the bifurcation of the aorta during a hysterectomy, without, however, leading to anatomic confirmation of the diagnostic hypothesis. This report emphasizes the need for detailed cardiological assessment of cases in which PVV are found on Doppler ultrasonography.^9^

Abbas et al.^10^ suggested the saphenofemoral junction compression maneuver as a supplementary method of differential diagnosis of pulsatility in varicose veins of the extremities. If pulsatility is interrupted after compression, cardiac etiology is more likely than arteriovenous communication.^10^

Although uncommon, varicorrhagia can cause voluminous bleeding because of the high venous pressure in these cases, which can be aggravated by anticoagulants taken by these patients, who often have comorbid atrial fibrillation.^2^ Considering the rarity and the associated high cardiac risk, definitive treatment of PVV remains controversial.^7^ Cases of venous insufficiency (VI) with mild symptoms appear to be adequately treatable with elastic compression and systematic elevation of the limbs.^8^

This strategy was effective for management of a case of PVV secondary to TR in a 55-year-old patient (CEAP C3EsAs,d,pPr) who refused surgical treatment for TR. With good adherence to clinical treatment of PVV, after 1 year of follow-up, symptoms improved and the treatment proposed was maintained.^11^

In patients with VI and associated complications, such as recurrent bleeding and chronic ulcerations, more invasive treatment may be needed.^2^^,^ 6 ^,^ 7 Casian et al.^7^ achieved complete ulcer closure and absence of relapses for 1 year after combined treatment of a patient with PVV. After failure of compressive clinical treatment, the patient underwent saphenous ligation at the saphenofemoral junction under local anesthesia, followed by retrograde catheterization of the great saphenous vein and infusion of 20 mL of thick foam, produced with the Tessari technique using 3% sodium tetradecyl sulfate solution. Despite full occlusion of the saphenous vein and its tributaries, the ulcer remained open after 3 months in follow-up, and treatment of insufficient perforating veins was performed by subfascial endoscopic ligature, with complete ulcer closure observed after 15 postoperative days.^7^

Chihara et al.^2^ reported their experience with treatment of PVV (CEAP 6) with recurrent bleeding in a patient with atrial fibrillation maintained on full anticoagulation with factor Xa inhibitor. The patient underwent endovascular laser treatment of the great saphenous below the knee while on anticoagulation. There were no hemorrhagic complications and the wound had healed by the 45th postoperative day without relapses over 6 months of follow-up.^2^ In turn, Badger et al.,^6^ decided to treat a patient with PVV (CEAP 4) with saphenectomy, 1 year after annuloplasty of the tricuspid valve.^6^

## CONCLUSIONS

Knowledge of the Doppler waveforms of peripheral flow is essential to understanding of changes secondary to several cardiac conditions, in addition to supporting the primary diagnosis. Given the complexity of the general status of patients with PVV secondary to TR, treatment should be chosen on a case-by-case basis as part of a multidisciplinary evaluation, taking into account the severity of clinical manifestations and the cardiovascular risk involved.
