# Supplementary material for: Pulsatile varicose veins simulating femoral artery aneurysm: case report
Source: J Vasc Bras. 2021 Jun 11;20:e20200070. doi: 10.1590/1677-5449.200070 (PMC8210542; doi:10.1590/1677-5449.200070)
Supplement: Translation

# Translation: Varizes pulsáteis simulando aneurisma de artéria femoral: relato de caso

**Authors:** Cleilson Almeida Marchesi, Márcia Porto Assis

**Affiliations:** 1 Universidade Federal do Espírito Santo – UFES, Hospital Universitário Cassiano Antonio Moraes – HUCAM, Vitória, ES, Brasil.

**Keywords:** varizes, pulsáteis, regurgitação tricúspide, ultrassonografia vascular

## Abstract

A regurgitação tricúspide severa é citada como fator associado ao desenvolvimento ou à recidiva de varizes em membros inferiores as quais podem, retrogradamente, apresentar um fluxo pulsátil. O diagnóstico etiológico diferencial desse achado ultrassonográfico deve ser feito com a pesquisa de fístulas arteriovenosas, tendo em vista as diferentes formas de tratamento. Dada a complexidade do estado geral dos pacientes com regurgitação tricúspide, a escolha do tratamento das varizes pulsáteis deve ser individualizada, fruto de uma avaliação multidisciplinar. Fazem parte do arsenal terapêutico todas as técnicas habitualmente empregadas no tratamento de varizes, bem como a associações entre elas a serem escolhidas levando-se em conta a gravidade das manifestações clínicas e o risco cardiovascular envolvido. Relatamos um caso de varizes pulsáteis secundárias a regurgitação tricúspide, diagnosticado durante a suspeita primária de aneurisma de artéria femoral em paciente de 73 anos, com a classificação clínica da doença venosa (CEAP) 4a, oligossintomática, tratada com medidas posturais e elastocompressão.

## INTRODUÇÃO

A prevalência das doenças vasculares periféricas tende a aumentar com a senilidade, sendo a coexistência com doenças cardiovasculares relativamente frequente^1^. A regurgitação tricúspide (RT) severa é citada como fator associado ao desenvolvimento ou recidiva de varizes em membros inferiores as quais podem, retrogradamente, apresentar um fluxo pulsátil^2^.

Embora o reconhecimento da pulsatilidade em veias periféricas em decorrência de RT e insuficiência valvar venosa esteja descrito desde 1827^3^, nos dias de hoje, mesmo com a ampla utilização do ultrassom Doppler, o desconhecimento do padrão dessas alterações se reflete na descrição pobre ou ausente delas nos laudos dos exames^4^. Relatamos um caso de varizes pulsáteis (VP) em paciente com RT, submetido à apreciação do comitê de ética (CAAE 36635420.1.0000.50.71), aprovado segundo o parecer 4.426.72.

## RELATO DE CASO

Paciente do sexo feminino, 73 anos, hipertensa, portadora de insuficiência cardíaca (IC) classe C e hepatopatia congestiva, atendida no pronto-socorro do hospital universitário com quadro de dispneia paroxística noturna e hemoptise. O ecocardiograma transtorácico evidenciou valva mitral espessada, com insuficiência importante e fluxo sistólico reverso em veia pulmonar esquerda superior; a valva tricúspide apresentava falha na coaptação com dilatação importante de átrio direito (56 mm) e de veia cava inferior (> 21 mm de diâmetro, com variabilidade < 50% à respiração).

Ao exame físico realizado no primeiro atendimento, notava-se edema simétrico de membros inferiores, varizes de grosso calibre, discreta dermatite ocre em terço distal de pernas, porém sem eczemas ou ulcerações [classificação clínica da doença venosa (CEAP) 4a]. Observou-se tumoração pulsátil notadamente em região inguinal esquerda.

Aventada hipótese de aneurisma de artéria femoral concomitante ao quadro varicoso, solicitou-se ultrassonografia Doppler arterial de membro inferior esquerdo, que mostrou fluxo trifásico em todos os segmentos estudados, sem alterações parietais significativas. Observou-se ausência de sinais ecográficos diretos e indiretos de comunicação arteriovenosa (fístula). Durante o estudo venoso, notou-se a ocorrência de fluxo alternante (bidirecional) em veia femoral comum e na junção safeno-femoral esquerdas. A complementação com o estudo venoso de ambos os membros não evidenciou sinais de trombose venosa profunda e demonstrou achado de curva espectral alternante também em eixo venoso profundo à direita, que se estendia por ambas as junções safeno-femorais e veias safenas magnas, bem como em dilatações varicosas no segmento de coxas. À esquerda, na topografia da tumoração em região inguinal, varizes de grosso calibre cruzavam anteriormente a artéria femoral, enquanto as tributárias varicosas que se estendiam pela coxa e joelho se comunicavam com veia perfurante de face medial da perna, no seu terço proximal, sendo que tal perfurante apresentava curva espectral alternante e fluxo direcionado, predominantemente, para o sistema venoso profundo (Figuras 1, 2 e 3).

**Figura 1:** Fluxo bidirecional pulsátil em veias femorais.

**Figura 2:** Fluxo bidirecional em junções safeno-femorais.

**Figura 3:** Fluxo bidirecional em veias varicosas de coxa esquerda e veia perfurante em perna.

Após o diagnóstico ultrassonográfico, foi solicitada avaliação conjunta pela equipe de Cirurgia Vascular, e esse exame físico revelou pulsatilidade em todo o eixo venoso superficial à ectoscopia, ausência de frêmito à palpação e enchimento lento de varicosidades à manobra de compressão/descompressão. Optou-se por tratamento clínico, com medidas posturais e compressão elástica. Ao longo da internação, a paciente apresentou melhora clínica significativa da cardiopatia e edema dos membros. Por se tratar de uma doença varicosa oligossintomática, comorbidade cardiológica grave recém-compensada e idade avançada, optou-se por manter o tratamento clínico ambulatorial, sem intervenções invasivas. Nos meses subsequentes, a paciente apresentou novos episódios de descompensação da IC e internações hospitalares recorrentes, evoluindo ao óbito após 1 ano de seguimento.

## DISCUSSÃO

Em condições fisiológicas, o fluxo venoso à ultrassonografia Doppler próximo ao coração mostra uma onda multifásica com dois componentes anterógrados e dois retrógrados. O primeiro e mais amplo componente anterógrado corresponde à pressão negativa do septo atrioventricular durante a sístole e é seguida por um componente retrógrado, que representa a pressão atrial positiva devido ao fluxo atrial. Uma nova deflexão positiva surge na abertura da válvula tricúspide seguida da última deflexão negativa produzida pela contração atrial^5^. À medida que se distancia do coração em direção aos membros inferiores, as altas complacências e capacitâncias desses territórios atenuam a pulsatilidade, e o fluxo assume aspecto fásico com a respiração^5^.

Na presença de RT grave, um fluxo pulsátil com componente retrógrado pode ser encontrado na veia supra hepática média e, mais raramente, em membros inferiores^5^. Segundo estudo de Ribeiro et al.^5^, a presença de pulsatilidade das veias femorais pode ser encontrada em cerca de 15,2% da população de maiores de 60 anos, tendo correlação importante com insuficiência tricúspide. Na presença de uma junção safeno-femoral incompetente, a “ventricularização” do fluxo do sistema venoso profundo decorrente de uma RT importante pode ser transmitida ao eixo safeno e a varicosidades de membros inferiores^1^^,^ 6 ^,^ 7. As alterações por efeito cardíaco no formato das ondas de fluxo dos vasos periféricos, na grande maioria das vezes, não são reconhecidas, e, quando o são, poucas vezes aparecem relatadas no laudo^4^.

A pesquisa de fistula arteriovenosa deve ser lembrada como diagnóstico diferencial no achado de pulsatilidade no sistema venoso, embora seja pouco provável quando o achado é bilateral^6^^,^ 8. Klein et al.^9^ relataram um caso de VP unilaterais que se desenvolveram 15 anos após plastia de válvula mitral com *bypass* cardiopulmonar intraoperatório. Nesse caso, o desenvolvimento do quadro varicoso ipsilateralmente à manipulação dos vasos femorais reforçou a hipótese de fístula arteriovenosa iatrogênica motivando a exploração cirúrgica da artéria femoral até a bifurcação da aorta durante uma histerectomia, sem, contudo, confirmação anatômica da hipótese diagnóstica. Tal relato corrobora a necessidade de avaliação cardiológica pormenorizada diante do achado de VP ao eco-Doppler^9^.

Abbas et al.^10^ sugerem a manobra de compressão da junção safeno-femoral como método auxiliar ao diagnóstico diferencial de pulsatilidade em varizes de extremidades. A interrupção da pulsatilidade após a compressão reforça a etiologia cardiogênica em detrimento das comunicações arteriovenosas^10^.

Embora infrequentes, varicorragias podem apresentar sangramento de volume importante pela alta pressão venosa nesses casos, condição que pode ser agravada pelo uso de anticoagulantes pelos pacientes que frequentemente apresentam fibrilação atrial como comorbidade^2^. Tendo em vista a raridade e o alto risco cardiológico associado, o tratamento definitivo das VP permanece controverso^7^. Casos de insuficiência venosa (IV) pouco sintomáticos parecem ser adequadamente tratados com elastocompressão e elevação sistemática dos membros^8^.

Essa estratégia se mostrou eficaz na condução de um caso de VP secundárias a RT em uma paciente de 55 anos (CEAP C3EsAs,d,pPr) que recusou tratamento cirúrgico da RT. Com boa adesão ao tratamento clínico das VP, após 1 ano de seguimento, houve melhora dos sintomas e manutenção do tratamento proposto^11^.

Na presença de IV com complicações associadas, como sangramento recorrente e ulcerações crônicas, um tratamento mais invasivo pode ser necessário^2^^,^ 6 ^,^ 7. Casian et al.^7^ obtiveram cicatrização completa e ausência de recidivas durante 1 ano após tratamento combinado de uma paciente portadora de VP. Após falência do tratamento clínico compressivo, a paciente foi submetida a crossectomia sob anestesia local, seguida de cateterização retrógrada de veia safena magna e infusão de 20 mL de espuma densa, produzida a partir de solução de tetradecil sulfato de sódio a 3%, sob técnica de Tessari. Apesar da oclusão completa da safena e de suas tributárias, em 3 meses de seguimento, a úlcera permaneceu aberta, sendo proposto o tratamento das perfurantes insuficientes por ligadura endoscópica subfacial, havendo cicatrização completa em 15 dias de pós-operatório^7^.

Chihara et al.^2^ relataram sua experiência com o tratamento de VP (CEAP 6) com sangramento recorrente em uma paciente com fibrilação atrial mantida em anticoagulação plena com o inibidor do fator Xa. A paciente foi submetida a tratamento endovascular com *laser* da safena magna abaixo do joelho em vigência de anticoagulação. Não houve complicação hemorrágica, e a cicatrização da ferida se deu no 45º dia de pós-operatório sem recidivas nos 6 meses de seguimento^2^. Badger et al.^6^, por sua vez, optaram pelo tratamento com safenectomia em uma paciente portadora de VP (CEAP 4) após 1 ano de anuloplastia da valva tricúspide^6^.

## CONCLUSÃO

O conhecimento dos padrões de ondas de fluxo periférico ao Doppler torna-se fundamental para o entendimento das alterações secundárias às diversas alterações cardíacas, além de subsidiar o diagnóstico primário. Dada a complexidade do estado geral dos pacientes portadores de VP secundárias à RT, a escolha do tratamento deve ser individualizada, fruto de uma avaliação multidisciplinar, levando-se em conta a gravidade das manifestações clínicas e o risco cardiovascular envolvido.
